# Treatment of Fracture of the Jaw, with Critical Remarks

**Published:** 1880-11

**Authors:** Thomas Brian Gunning

**Affiliations:** New York


ARTICLE VI. PART VI.

### TREATMENT OF FRACTURE OF THE JAW, WITH CRITICAL REMARKS.

BY THOMAS BRIAN GUNNING, D. D. S., NEW YORK.

The following case shows that recourse must sometimes be had to the surgeon's knife. In the terrible stage accident at the White Mountains in August, 1873, when so many passengers were killed and injured, one received a compound fracture in the lower jaw, between the right canine and the lateral incisor teeth, and for several days remained insensible through concussion of the brain. Professor Charles E. Buckingham of the Medical School of Harvard University had charge of this patient, assisted by physicians of the vicinity, and a dentist who had attended the Philadelphia Dental College. On the sixteenth day, Dr. T. M. Cheeseman, of New York, was called in consultation, but as the surgeon in charge expressed the opinion that it was safe to leave the jaw without any mechanical appliance, Dr. Cheeseman retired from the case; and through his advice it came into my hands on September 3d, the patient bringing letters to me from both surgeons. Dr. Buckingham said that three separate attempts had been made to force the fragments up into place by bandages, but pain in the left articulation and masseter muscle was so severe that the bandages were given up. He had thought it best to leave the whole to itself, that nature would do the best for this particular patient. The fracture was now firmly united.

The gentleman also brought a hard rubber splint made for the chin and jaw, and two small caps for molar teeth. I found his front teeth down five-sixteenths of an inch below the right canine, the wisdom teeth a full quarter of an inch too close together, and the tonsils which lie inside the angles of the jaw still closer.

The lower right canine was the only tooth which touched the upper when the jaw closed, and this tooth, with those behind it, being so much above the front incisor teeth, in fact, all in the left fragment, the mouth was not only unsightly but the teeth useless in eating; while the tongue was restricted by the closeness of the angles of the jaw. This condition was evidently brought about through muscular inaction of the parts during the patients' insensibility, when by lying on his side probably, the left fragment holding ten teeth was pressed in and the back ones impinging against the upper back teeth, threw the front of the jaw down below the right fragment in which position they united. After this, attempts to force the parts up by bandages, of course failed, and as time went on the union grew stronger until the case came under my treatment about twenty-four days after the accident.

The patient was but twenty years old, and his jaw having united so strongly, it was clear that if broken again and set right, the bone would again unite; therefore, after taking wax impressions of both upper and lower teeth, I cut the bone apart as follows. A powerful gum lancet was passed through the new growth at the junction of the lip with the front of the lower jaw and one upward cut separated all above; then the sides of the jaw were pulled apart, the left by an assistant; this separated the canine and the lateral incisor teeth enough to allow the knife to stand vertically between them, and from this position the point of the lance gradually cut down through the new growth until the fragments of the bone were so loosened from each other that the teeth in both were easily brought to the same level.

The splint should have been put on while the patient was insensible, but as the surgeons left the fracture until it united, they should have cut it apart before they applied the bandages, in order to allow the bone to go into place.

I found it necessary to cut the fragments apart before making the splint, to find out whether they could be brought precisely into their original relations. A plaster cast can be sawed apart precisely where the jaw is fractured, but if the parts of the bone cannot be put back exactly, the cast should also deviate as the splint must be made to fit. These two fragments, however, came into exactly the same relations, and a splint covering all the teeth and fastened by screws to the left canine and right first molar, was worn for thirty days; the screws were then left out and the teeth filled, but the splint worn thirty days longer, although removed at pleasure; the jaw was then strong and in its old place and was used in eating and speaking throughout the treatment.

No trace of the separation by the knife was seen, except a fine line on the gum, as I had taken care not to cut into the lip, nor into the soft parts under the tongue, and not to allow the point of the lancet to touch the skin after it had passed through the whole depth of the jaw.

Had the break between the bone passed off to either side, that is, had it, instead of being straight, turned from its first direction, a bent blade could have followed unless the angle were very sudden. If a fracture were bent too abruptly to be followed, and it could not be wrenched apart, it should be sawed off on a line with the first part of the fracture; a small saw could be used with a dental engine. But if an opening in the skin were required it should be cut under the border of the jaw so as not to appear in front.

Certainly no such operation can now be justifiable as that quoted by Professor Hamilton as follows:

"In the case of the fracture of the inferior maxilla, reported by Dr. Buck, to the New York Pathological Society, and already referred to, the bone was broken between the two incisor teeth of the left side; the part of the bone on the left of the fracture was driven in, and interlocked behind the end of the right portion, so as to be separated by a finger's breadth. Finding it impossible otherwise to reduce the fracture, Dr. B. dissected off the under lip, so as to expose the fracture. He found that the right anterior portion of the fractured bone terminated in an angular projection as far as on a line below the left angle of the mouth. The lip was then divided to the chin, and the soft parts holding the fragments together incised. A chisel was then insinuated behind the projecting angle of the bone, while it was being excised by the metacarpal saw. When the bone was restored to its natural position, it was found so apt to be displaced, that holes were drilled at the lower angle of the fracture, and adjustment maintained by wiring them together, the wire passing out through the lower angle of the wound. Sutures and adhesive straps, with a bandage, were employed to maintain the adjustment of the parts. So far, the patient has done well, being supported by liquid nourishment introduced through a tube, passed through the space left by one of the incisors, which, on account of its looseness, was removed.*

In many cases much trouble is experienced in attempting to set fractures of the lower jaw *after adhesions* have set in.

In the case of C. B., in which so much trouble was experienced by the surgeons in Brooklyn, I feel assured that the *springing* of the

*Opus cit. p. 118. Also New York Journal of Med., &c., March, 1847, p. 211.

jaw back and the angles out was kept up by the early union of the soft parts around the fracture. The man was young and robust, and as the swelling of his neck pushed the angles out and his strong muscles drew in the front of the jaw, adhesion set in and held the fragments which were well in place at the fractured surfaces, except having a little too much new growth between the inner part of the bone. This *elastic* union in and through the socket of the right canine tooth resisted all their attempts to hold the angles of the jaw in, and bring the front of the jaw forward. On the second day the parts were sore and the man too weak, and as he became better able to bear pain, the union of the soft parts grew stronger, and resisted the misdirected efforts of the surgeons.

Notwithstanding these proofs of "immediate union," (Dr. Macartney's term,) in the soft parts, it is clear that the surfaces of these ragged wounds do not fit closely but in some of their fibres require lymph or other intervening substance and unite more gradually; never so quickly as in a case of immediate union, completed in four or five hours.*

In compound fractures where the fragments of the bone and their surrounding tissues move freely, this inconvenient union or adhesion of misplaced parts is not found, except in old injuries.

These views upon the union of the tissues which surround and cleave to the fractured bone are in accordance with those of standard writers on pathology. I cannot, however, agree with them so far as to admit in respect to the maxillary bone that there is so long a period of delay of the reparative process as they suppose to be usual in compound fractures of human bones. Mr. Paget thinks that the period of inflammation with seeming inaction and recovery, *previous* to the production of reparative material to be from eight to ten days.†

Now, with the fragments of the jaw moving, and their fresh broken surfaces out of sight, no judgment can be formed of the process of repair. But when, as in case 2, the parts were tied together in an hour and a half from the fracture and did not again separate; while in C. B.'s case the fragments united so soon, and in view of the firm union by the 24th day of the fracture of the White Mountain case;

*Lectures on Surgical Pathology, by James Paget, F. R. S., D. C. L., Oxon. Third Edition, p. 144. Philadelphia, 1871.

†Opus cit. p. 193.

I must claim for the jaw an exceptional position and place it, especially when promptly controlled by a splint, in the category of those where union commences very soon, as in animals.

Case 2 gives a brief statement of a fracture in my own jaw, in which after the ligatures had held the fragments for several hours, the parts were then controlled by the thumb and fingers four and a half hours more, when the splint was applied, and worn when the jaw had been held *in all*, twenty hours. The splint was then taken off, but not with difficulty, as it fitted tightly, and after a hole was sunk in each first molar, it was again put on and screwed fast. During all this I felt nothing like movement of the fractured surfaces. Yet the fragments were by the accident, only 21½ hours before, displaced to a very great extent, and the soft parts much lacerated. They, however, were soon placed exactly together, and the splint covered them so, that with the saliva, etc., the fracture was shut in completely from the air; while the splint by means of the teeth held the bone in place at once firmly, much sooner and more firmly than the ensheathing or provisional callus so early formed around the fractures of the lower animals; but which in man is rarely formed except in fractures of the ribs, where movement cannot be entirely prevented; and, as Mr. Paget thinks, perhaps in the long bones of young children.

It is thus shown that when a splint is properly applied to a fractured maxillary bone with the gum and periosteum torn open, this compound fracture is at once placed virtually in the condition of a simple fracture, and that the splint, by means of the teeth, holds the fractured surfaces of the bone precisely and firmly in place against each other. Referring to such rare cases Professor Paget says:

"The union o fracture is commonly affected by the organization of new material connecting the fragment. Sometimes, indeed, immediate union occurs. When portions of the bones are placed and held in exact apposition, they may be united without any new material being formed for their connection; a continuity of tissues being restored, as in the cases of healing by immediate union of soft parts. But this is rare, and has not yet been sufficiently studied."*

Thus, what was rare to Professor Paget, my splint has made easily attainable and frequently accomplished in respect to the upper and

*Opus cit. p. 181.

lower maxillary bones; the last mentioned being, next to the ribs, the bone most imperatively called into service.

In the prompt setting of my own jaw, and early application of the splint, the first ever used without being supplemented by external appliances, there being consequently nothing to press upon the soft parts, nor impede the nervous and vascular circulation, nor limit the muscular action I found, as I thought, that the bone had within itself and the tissues which cleave to it, reparative power which at once set to work to repair its fracture. In the paper which I read before the New York Academy of Medicine on June 1st, 1864, after fully describing the case, I said:

"After making due allowance for the interlacement of the fragments, would the bone have held together, unless assisted by some reparative process even then going on?"

The experience of sixteen years which have passed since I asked that question, has confirmed me in the opinion that repair in the human jaw bone commences as early as in the soft parts which surround it.

This conviction has been forced upon me by the results of treatment in fractures of the jaw under the use of interdental splints, which reflection assures me were the simple consequences of efficient assistance by which the *natural processes* of repair were protected from interference.

I believe, also, that could the other bones be held as well, that the healing processes would set in very early.

These remarks are not intended to intimate that I claim to have made any progress through personal observation of healing processes beyond the discovery that they are simultaneous in the bones and in the tissues cleaving to them.

I have otherwise simply learned from able and indefatigable men whose writings have so enlarged our knowledge of physiological processes and pathological conditions. It would hardly be decorous to them or just to myself to leave this unsaid.

PROGNOSIS depends so much upon the treatment that I deferred speaking of it until some remarks upon the latter should in a measure prepare the way for its consideration.

M. Malgaigne's section upon prognosis, in fractures generally, precedes that on treatment, but his remarks clearly justify my arrangement and they are, while concise, so comprehensive and withal so fitted to my purpose that in a later page I quote the opening and closing sentences of the section. To take more would be unjust to the translator. Malgaigne's admirable work published in 1847, was translated into English in 1859, and Professor Paget's "Lectures on Surgical Pathology," from which I also quote, were printed in 1853, and repeated editions have followed. Now, with such great sources of information open to all engaged in Medicine and Surgery, when giving my views in 1866, upon the treatment of Fracture of the jaw by interdental splints fitted accurately to all the teeth of the broken bone, I merely said in respect to their effects.* "These splints hold the fragments so well together that I have seen badly lacerated gums heal up in from two to three days, so perfectly that the fractures were then only simple.

"No bad effects are produced by splints covering the teeth and gum. On the contrary, teeth that are so much loosened by the injury as to be beyond recovery in the usual treatment, are securely held by the splint and become firm again. The gum looks red and soft while the splint is worn, but a short period suffices for its complete restoration, even when it has been covered up for months. I generally leave the splint on long enough to feel assured that temporary removal will not endanger the union, which is very delicate for some time. How soon this will be, after the first application of the splint, and how long before the splint can be dispensed with, depend upon the gravity of the injury, and the state and the age of the patient."

These explanations seemed at the time quite sufficient, especially as they were complemented by cases carefully selected to illustrate the use of the several splints and also to show the results which had been observed in the six years since the first splint was called into use through the explosion on a Spanish frigate. These results of treatment were the surest guide to the surgeon in his prognosis on these injuries when treated on the same system, and on his comparison with those treated by other methods.

For twelve years nothing appeared to raise the belief that any well informed practitioner, even if without personal observation of the splints in treatment, would be unable to comprehend and estimate those pathological conditions which they were devised to meet, so

*New York Medical Journal, Vol. III, p. 447.

far, at least, as to judge intelligently to what extent a patient's condition admitted of relief by surgical art. But in March, 1879, an article was published in the New York Medical Journal, which indicated a disregard of fair consideration of the pathological conditions and of the facts involved, and which in other respects required correction. The publishers, Messrs. D. Appleton & Co., in replying, said:

"We write to-day further, that not being members of the profession, it is impossible for us to form any opinion of the matter in dispute, or to judge of the merits of any article on a medical topic." They, however, promised a correction by the editor, and the following appeared in the May number of the journal.

"Correction.—On page 281, of our March number, statements were inadvertently admitted, which disparaged the treatment of the late William H. Seward, whose injury, it will be remembered, was so severely complicated by the subsequent attempt to assasinate him."

JAMES B. HUNTER, M. D., *Editor.*

At the request of the publishers, this statement was drawn up by me, and upon their pleading disability, I suggested the "Medical Editor," instead of the publishers, should make the correction; in his correction, however, as above, the word "*unjustly*" was omitted before the word "*disparaged*" as well as the words "*Dr. Gunning's*" after it, and the following sentence of my statement was entirely ignored, viz.: "The report of this case first appeared in Vol. IV, of this journal, and shows conclusively that Dr. Gunning's treatment was eminently successful." Thus Dr. Hunter further misled the readers of the journal by calling attention to the false statements without adequately correcting them.

These statements are now quoted, viz.:

*"The late Wm. H. Seward, when traveling around the world, and when at Tokohoma, Japan, required the services of a dentist. Upon examination it was found that the inferior maxilla was comparatively useless for masticating purposes, there being a false joint at the seat of the original fracture, no union having taken place. This case will be remembered from the world wide notoriety of the circumstances attending the injury, as well as the reports, which have been universally believed that the patient was benefited by the treatment he received for the cure of his fracture."

*New York Medical Journal, Vol. XXIX, p. 281.

It is thus intimated that Secretary Seward's jaw was broken only in one place, and that my report, founded on personal observation of the case, and the subsequent statements of the patient, which led to the belief that he was benefited by my treatment, were false.

The report, case 8, was quite circumstantial, and it shows that there were two fractures in the jaw, that I set them and applied the splints, that one united by bony union, and the fibrous union of the other, grew firm enough to do without a splint, and continued to improve so that in March, 1866, Mr. Seward wrote: "Thus at last I begin to regard my cure in that respect complete." The report was first published in the "New York Medical Journal," when edited by Doctors Hammond and Dunster, the predecessors of Dr. Hunter; and it remained uncontradicted until the article was published, entitled "Gun-Shot Wounds of the Mouth;" which contained the slanderous reference to Mr. Seward's case. His case having no relation to the subject of the paper nor to the case put in comparison with it. Dr. Hunter in alleging oversight, gave no reason for the singularly limited wording of his "Correction," nor is it necessary to suggest any at this time, except to admit that it may have arisen in part from inability to comprehend the very serious character of Mr. Seward's case. But as editor for 8 or 9 years, he must be held to know of much that has been written by Howship, Stanley, Malgaigne, Paget, and many other observers of pathological conditions and results. It is thus proper to assume that Dr. Hunter's difficulty was in the unparalleled complications which surrounded Mr. Seward's case. As many have been misled by the improper course pursued in respect to his treatment, it is advisable to explain its special difficulties and make them clear.

The report of 1866 is quite full, but the time which has since passed, while showing the necessity of its being supplemented, has also cleared the way for some instructive explanations which could not in the first report be entered into. Now, however, it is proper to make them in order that surgeons and physicians may have the fullest experience of the past to aid in prognosis in future cases, and at the same time show them that the treatment by interdental splints effectually controlled Secretary Seward's fractured jaw.

(*TO BE CONTINUED.*)

